# Using community level strategies to reduce asthma attacks triggered by outdoor air pollution: a case crossover analysis

**DOI:** 10.1186/1476-069X-13-58

**Published:** 2014-07-11

**Authors:** Loren H Raun, Katherine B Ensor, David Persse

**Affiliations:** 1 Department of Statistics, Rice University, 6100 Main Street, Houston, TX 77005, USA; 2 City of Houston Health and Human Services Bureau of Pollution Control and Prevention, 7411 Park Place Blvd, Houston, TX 77087, USA; 3 City of Houston Emergency Medical Services, 600 Jefferson Suite 800, Houston, TX 77002, USA; 4 Department of Medicine, Baylor College of Medicine, One Baylor Plaza Houston, Houston, TX 77030, USA

**Keywords:** Asthma, Air pollution, Risk, Ozone, Nitrogen dioxide, Action plans

## Abstract

**Background:**

Evidence indicates that asthma attacks can be triggered by exposure to ambient air pollutants, however, detailed pollution information is missing from asthma action plans. Asthma is commonly associated with four criteria pollutants with standards derived by the United States Environmental Protection Agency. Since multiple pollutants trigger attacks and risks depend upon city-specific mixtures of pollutants, there is lack of specific guidance to reduce exposure. Until multi-pollutant statistical modeling fully addresses this gap, some guidance on pollutant attack risk is required. This study examines the risks from exposure to the asthma-related pollutants in a large metropolitan city and defines the city-specific association between attacks and pollutant mixtures. Our goal is that city-specific pollution risks be incorporated into individual asthma action plans as additional guidance to prevent attacks.

**Methods:**

Case-crossover analysis and conditional logistic regression were used to measure the association between ozone, fine particulate matter, nitrogen dioxide, sulfur dioxide and carbon monoxide pollution and 11,754 emergency medical service ambulance treated asthma attacks in Houston, Texas from 2004-2011. Both single and multi-pollutant models are presented.

**Results:**

In Houston, ozone and nitrogen dioxide are important triggers (RR = 1.05; 95% CI: 1.00, 1.09), (RR = 1.10; 95% CI: 1.05, 1.15) with 20 and 8 ppb increase in ozone and nitrogen dioxide, respectively, in a multi-pollutant model. Both pollutants are simultaneously high at certain times of the year. The risk attributed to these pollutants differs when they are considered together, especially as concentrations increase. Cumulative exposure for ozone (0-2 day lag) is of concern, whereas for nitrogen dioxide the concern is with single day exposure. Persons at highest risk are aged 46-66, African Americans, and males.

**Conclusions:**

Accounting for cumulative and concomitant outdoor pollutant exposure is important to effectively attribute risk for triggering of an asthma attack, especially as concentrations increase. Improved asthma action plans for Houston individuals should warn of these pollutants, their trends, correlation and cumulative effects. Our Houston based study identifies nitrogen dioxide levels and the three-day exposure to ozone to be of concern whereas current single pollutant based national standards do not.

## Background

Asthma is a serious and sometimes life-threatening chronic respiratory disease that affects almost 25 million Americans and costs the nation $56 billion per year [1]. In 2009, 3.3 deaths per 100,000 people were attributed to asthma and there were 1.9 million asthma related emergency department visits [2,3]. Asthma prevalence increased from 7.3% in 2001 to 8.4% in 2010, when 25.7 million persons were diagnosed with asthma [4].

Although the association between air pollutants and asthma attacks is well documented [5,6], the lack of specific guidance in asthma intervention programs to reduce exposure beyond broad nationally set air quality alerts may severely limit effectiveness of the air quality alert approach. In a recent review of the literature two effective intervention methods were identified, namely self-management education and more general comprehensive home-based multi-trigger reduction interventions [7]. However, in their umbrella review the authors were unable to identify any reviews related to the effectiveness of alerts for air quality.

Currently air quality alerts in the United States address pollutants in isolation from each other, but individuals are exposed to a mixture of pollutants. A barrier to pollution specific asthma education is that detailed pollutant-asthma guidance is contingent upon the development of methods to address multi-pollutant mixtures [4]. Asthma attacks are triggered by multiple Environmental Protection Agency (EPA) criteria pollutants, namely ozone (O_3_), nitrogen dioxide (NO_2_), particulate matter (PM), sulfur dioxide (SO_2_), and carbon monoxide (CO) [8-12]. An additional complication is that cities have different mixtures of these pollutants. The need to address multiple pollutants in the criteria pollutant review and standard setting process was identified in 2004 [13]. Researchers focused on developing national criteria pollutant standards in the multi-pollutant exposure context have made limited progress over the last decade [14-19]. Until multi-pollutant statistical modeling fully addresses this gap, more specific guidance to mitigate risk from exposure to pollutants is required. One solution is to develop guidance on a city-specific basis, especially in highly polluted cities.

To demonstrate the city-specific guidance, asthma risks were evaluated using an environmental public health tracking model framework [20] for Houston, Texas. With this model, our focus is on tracking the “association” within a city. The concern is the pollutant mixture, not the individual pollutants. Moreover, regular tracking of the association within a city can be used to evaluate trends reflecting the effectiveness of regulatory measures, interventions, or identify changing potency of air pollutants [20].

Houston is the fourth largest city in the United States and has recognized air pollution problems [21]. The Houston-Galveston region has an extensive air-monitoring network. Furthermore, Houston emergency medical service (EMS) responded to 11,754 emergency calls for asthma from 2004 through 2011. Each EMS response cost approximately $1,400 for a total estimated cost of $17 million [22]. Currently, asthma action plans vary in Houston. Some plans address only medications and lung function and others extend to a check box of asthma symptom triggers. For example, the Houston Independent School District asthma action plan includes air pollution as an asthma symptom trigger but more detailed information is needed regarding which pollutants are of concern, their trends, correlation, and cumulative effects.

A case-crossover analysis was used to measure the association between asthma associated criteria pollutant levels and EMS calls for asthma attacks from 2004 to 2011 in Houston. The pollutants examined were daily ozone, PM with an aerodynamic diameter less than 2.5 microns (PM_2.5_), NO_2_, SO_2_ and CO. In our analysis we first developed single, and then multi-pollutant models of the association. We also segmented the overall model by time to examine trends and by demographics to examine effect modification. We then analyzed the association based on ranges of concentration to formulate concentration-risk curves. This analysis is followed by a discussion of recommendations for asthma action plans for asthmatics in Houston. This research uncovers some critical new data that may be helpful in developing guidance on a city-specific basis.

## Methods

### Study design and setting

The data used in this study were obtained from the Houston Fire Department EMS call database segmenting by two fields, working assessment and treatment. The selection for working assessment was asthma and for treatment administered was nebulized albuterol (n = 11,754). The working assessment input is determined by EMS personnel and identifies the primary reason for treatment. The data were obtained during the eight-year period (2004-2011). Rice University and Baylor College of Medicine Institutional Review Boards approved all data-collecting procedures for human subjects.

### Participant data

Included in the study were all patients older than two years of age. Patients two years and younger were excluded from the study because the diagnosis is less reliable. If EMS responded to the same person multiple times within two weeks, the first call was retained and subsequent calls were removed from the database for analysis [20,23]. There were no other exclusion criteria. The EMS database consists of data collected according to National EMS Information Systems guidelines [24]. In addition to recording the working assessment and the administration of albuterol, the database also includes the following relevant information: time of call, location, age, sex, and race of patient.

### Ambient air quality, meteorological, and other data

Ambient pollution concentration data were obtained from the Texas Commission of Environmental Quality (TCEQ). In this analysis, hourly data from 35 ozone, 13 NO_2_, nine CO, nine PM_2.5_, and eight SO_2_ monitors in the Houston Metropolitan Area were used. The daily average values of ozone, NO_2_, CO, PM_2.5_, and SO_2_ were calculated across monitors. Researchers commonly use the average concentrations across monitors to obtain one average pollution level in case cross-over analysis [25-28]. The use of the average, over other spatial exposure estimation methods (e.g, inverse distance or kriging), is preferred when the activity patterns of the subject are not known or cannot be reasonably assumed to be similar on case and control periods.

The daily maximum 8 hour running mean was also calculated for ozone. The number of air monitors measuring a specific pollutant changed through the study years as monitors went on and off line. However, more than 99% of the time at least one monitor was operating for each pollutant. All air pollution data were collected using EPA federal reference methods [29] and validated by the TCEQ.

Ambient apparent temperature was used to control for meteorological conditions. The apparent temperature was calculated with the method used by O’Neill et al. [30] originally described by Steadman and Kalkstein and Valimont [30-32]. Aeroallergen data available for the study area are in the form of daily pollen and mold spore counts collected by the Houston Department of Health and Human Services at a single location using a Burkard Spore Trap sampling at 10 liters/minute [33]. During the study period, these data were largely incomplete. The percent of complete daily tree, grass and weed pollen data was 42.1%, 53.5% and 61.0%, respectively, and the percent of complete daily ascomycetes and basidiomycetes spore data was 61.0% and 62.6%. Therefore, these data could not be included in the analysis. Weeks with reported influenza and major U.S. holidays were flagged with an indicator and incorporated in the model [31].

### Statistical analysis

The data were analyzed using a time-stratified case-crossover design coupled with conditional logistic regression [34]. All tests are conducted at a significance level of 0.05. The case-crossover design was first introduced by Maclure (1991) and is increasingly used in studies to assess episodic events following short-term exposure to air pollution [25-27,35-39]. In the case-crossover design each individual experiencing a health event serves as his or her own reference, in other words, individuals act as their own control. Ambient air pollution was used as a proxy for personal exposure. The ambient air pollution concentrations at times when the study individual is not experiencing the asthma attack are the reference concentrations. Referent exposures, selected by time stratified sampling, were the exposures on all days falling within the same month and on the same day of the week as the event [40]. This reference period design has been shown to limit bias caused by patterns in air pollution [40]. Conditional logistic regression was applied to estimate the association of pollution and increased relative risk of the health event while controlling for confounding factors.

Following exploratory data analysis, the association of EMS calls for asthma attacks and the potential confounding variables (apparent temperature, holidays and influenza season) was examined. The form and lags of these variables showing the strongest association with EMS calls for asthma attacks according to the lowest Akaike Information Criterion (AIC) score were included universally in the pollution models. All further modeling included the confounding variables.

Sensitivity analysis with pollution lag models was conducted to examine the association of single air pollutants and asthma attacks. The association at the day of onset (lag 0 day), one to three days prior to onset (lag 1, 2, 3) and constrained distributed lag models (0-1 day, 1-2 day, and 0-2 day) were examined.

Significant associations found in the exploratory single pollutant analysis were combined in a multi-pollutant model. Interaction terms were also explored. Regression diagnostics were used to define the final multi-pollutant model. The final multi-pollutant model was used to examine stratification, time segments, and concentration-risk curves. The case-crossover logistic regression was conducted in SAS version 9.3 [41].

## Results

### Exploratory data analysis

A breakdown of the EMS calls for asthma attacks occurring during the study period are presented in Table 1 by age group, sex, race, season and year. The age group of patients less than 24 years old comprises of 31% of the calls. Of the remaining 69% of patients 27% fall in age group 25 to 45, 29% in age group 46 to 66 years, and those 67 and above comprise 13% of the sample. There are approximately 9% more female patients compared to male and the predominant race of patients is African American. The statistics of those requiring EMS ambulance treatment for asthma attacks are consistent with Center for Disease Control statistics on asthma prevalence based on data from 2008 to 2010 [4]. Generally, there were fewer calls in the cold season than the warm.

**Table 1:** Number of EMS-treated asthma attacks by age group, sex, race, season and year

**Characteristic**	**No. of Calls**	**%**
Age (in years)		
<24	3,686	31.4
25-45	3,146	26.8
46-66	3,402	28.9
≥ 67	1,520	12.9
Sex		
Female	6,422	54.6
Male	5,332	45.4
Race		
Asian	173	1.5
African American	8,233	70.0
Caucasian	1,761	15.0
Hispanic	1,475	12.5
Indian	63	0.5
Unknown	49	0.4
Season		
Warm (April to October)	6,519	55.5
Cold (November to March)	5,235	44.5
Year		
2004	1,203	10.2
2005	1,368	11.6
2006	1,354	11.5
2007	1,660	14.1
2008	1,665	14.2
2009	1,464	12.5
2010	1,544	13.1
2011	1,496	12.7
Total	11,754	100

**Figure 1:**
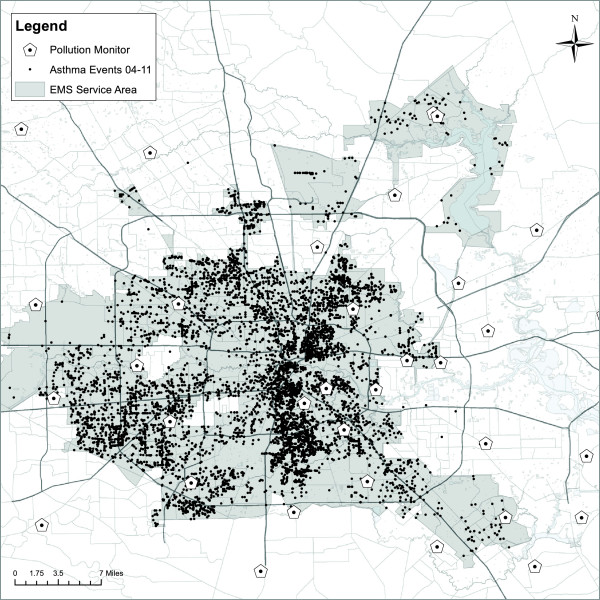
EMS-treated asthma attacks and pollution monitors in Houston, Texas (2004-2011).

**Figure 2:**
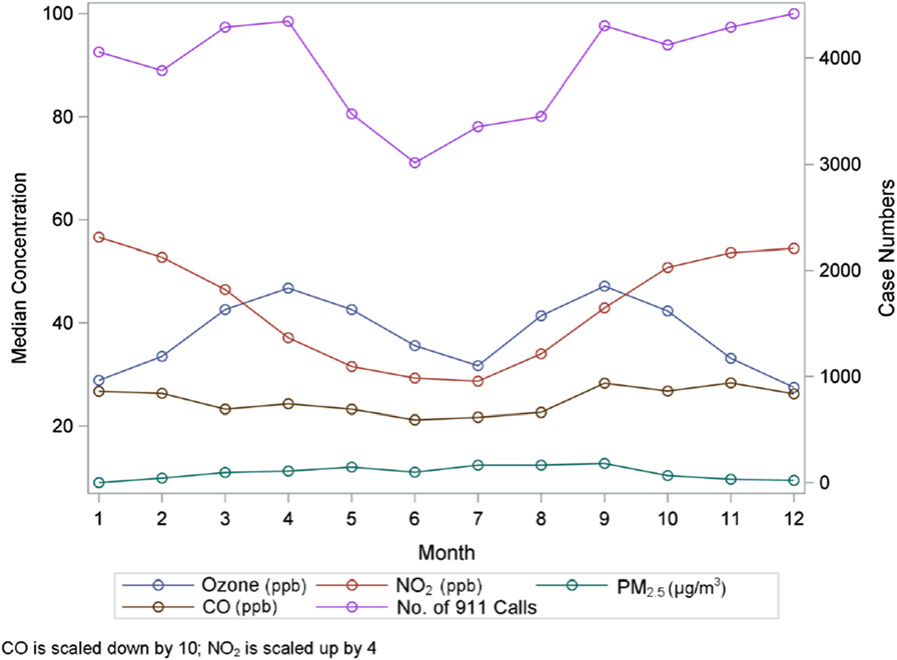
Number of EMS-treated asthma calls in Houston by month and median pollution concentration (2004-2011).

Daily levels of the pollution and meteorological data are presented in Table 2 by all year and season. Less than 1% of pollution and meteorological data were missing during the study period. In general, NO_2_ and CO appear higher in the winter than summer while the opposite is true for PM_2.5_ and ozone. The locations of the air monitors used in the study in relation to the EMS-treated asthma attacks are shown in Figure 1. The median concentrations by month over all years and all monitors are plotted with the monthly counts of EMS-treated asthma in Figure 2. Ozone and NO_2_ concentrations dip in June and July as do the number of EMS cases. In addition to the median, July has the lowest frequency of days when the maximum eight hour average concentration of ozone met or exceeded 76 parts per billion at a monitor (not shown) [42]. These lower ozone concentrations in June and July coincide with high daily rain frequency in these months [42]. Pearson correlation coefficients between daily measures of air pollutant concentrations and apparent temperature indicate the strongest correlations between daily pollutants were between NO_2_ and CO (r = 0.74) followed by NO_2_ and SO_2_ (r = 0.57), by CO and SO_2_ (r = 0.56), daily PM_2.5_ and ozone (r = 0.42). The correlation between ozone and NO_2_ was (r = 0.23). The strongest correlation between a pollutant and apparent temperature was for NO_2_ (r = -0.54). As discussed below, the interaction terms between model variables were not significant.

**Table 2:** Daily pollution and meteorological levels 2004 to 2011

	**All Year**				**Warm (April to October)**				**Cold (September to March)**			
		**Percentile**				**Percentile**				**Percentile**		
	**% Complete**	**25th**	**50th**	**75th**	**% Complete**	**25th**	**50th**	**75th**	**% Complete**	**25th**	**50th**	**75th**
CO (ppb)	99.9	186.8	246.5	336.9	99.8	178.6	239.1	318.0	100.0	198.8	261.0	374.5
NO_2_ (ppb)	99.8	7.3	10.5	14.5	99.7	6.1	8.9	12.2	100.0	9.7	13.2	17.1
O_3_ 8 hr (ppb)	100.0	27.5	35.8	48.1	100.0	29.6	40.6	55.4	100.0	25.6	32.1	39.4
PM_2.5_ (μg/m^3^)	99.8	8.4	10.7	14.1	99.7	9.1	11.7	15.5	100.0	7.6	9.7	12.3
SO_2_ (ppb)	99.9	0.7	1.5	2.5	99.8	0.6	1.2	2.2	100.0	1.1	1.9	2.8
Temperature (°C)	100.0	16.5	22.7	27.6	100.0	24.0	26.9	28.9	100.0	11.1	15.4	19.4
Relative Humidity (%)	99.9	62.4	70.3	77.2	99.8	63.5	69.4	74.5	100.0	59.3	72.6	80.8
Apparent Temperature (°C)	99.9	15.5	24.5	31.9	99.8	26.1	31.0	33.7	100.0	8.9	14.0	20.2

### Case-crossover and conditional logistic regression analysis

Analyzing the association between asthma and average apparent temperature using the conditional logistic regression model showed that the previous day was the relevant exposure period. The minimum AIC was used to select the best model. A similar study found the same result [20]. The logistic regression assumption of linearity in relative risk is appropriate in this case. In addition, controlling for holidays slightly increased the relative risk of an asthma attack while influenza season had no effect. Again, these results were found by other researchers, with confounding from asthma attacks around the holidays given in [43] and the non-effect of influenza reported in multiple studies [43-46].

Results of the single pollutant exploratory analysis lag models are shown in Table 3 where we list the adjusted relative risk of EMS calls for asthma attacks from exposure to an increase in interquartile range (IQR) of the respective pollutants. The lag for the statistically significant relative risk model with the minimum AIC is indicated in Table 3 with an asterisk (*).

**Table 3:** Analysis of relative risk for EMS-treated asthma attacks per IQR increase in single pollutant

			**All Year**		**WARM**		**COLD**	
					**April to October**		**November to March**	
**Pollutant**	**Lag**	**IQR**	**RR**	**95% CI**	**RR**	**95% CI**	**RR**	**95% CI**
NO_2_ (ppb)	0 Day*	8	1.12	1.07-1.17	1.08	1.00-1.16	1.14	1.08-1.20
	1 Day		1.04	1.00-1.09	1.02	0.95-1.10	1.05	1.00-1.11
	2 Day		1.03	0.99-1.07	1.06	0.99-1.13	1.01	0.96-1.06
	3 Day		1.02	0.98-1.06	1.05	0.99-1.12	1.00	0.95-1.05
	0 to 2		1.10	1.04-1.16	1.07	0.98-1.17	1.10	1.03-1.18
CO (ppb)	0 Day*	155	1.05	1.02-1.08	1.03	0.98-1.08	1.06	1.03-1.10
	1 Day		1.03	1.00-1.05	1.00	0.95-1.05	1.04	1.00-1.07
	2 Day		1.02	0.99-1.04	1.03	0.98-1.08	1.01	0.97-1.04
	3 Day		1.01	0.98-1.04	1.03	0.99-1.08	1.00	0.96-1.03
	0 to 2		1.04	1.01-1.08	1.01	0.96-1.07	1.06	1.01-1.10
O_3_ (8 hour) (ppb)	0 Day	20	1.04	1.01-1.07	1.01	0.98-1.05	1.11	1.04-1.18
	1 Day		1.06	1.03-1.09	1.02	0.99-1.06	1.16	1.09-1.24
	2 Day		1.05	1.01-1.08	1.03	0.99-1.07	1.07	1.01-1.15
	3 Day		1.01	0.98-1.04	1.01	0.98-1.05	0.99	0.93-1.06
	0 to 2*		1.07	1.03-1.11	1.03	0.99-1.08	1.17	1.08-1.27
PM_2.5_ (μg/m^3^)	0 Day*	6	1.02	0.99-1.05	1.03	0.99-1.06	1.00	0.95-1.06
	1 Day		1.02	0.99-1.05	1.03	0.99-1.06	1.00	0.95-1.05
	2 Day		1.04	1.01-1.07	1.03	1.00-1.07	1.06	1.01-1.12
	3 Day		1.03	1.00-1.06	1.03	1.00-1.07	1.01	0.96-1.06
	0 to 2		1.04	1.00-1.07	1.04	0.99-1.08	1.03	0.97-1.10
SO_2_ (ppb)	0 Day	2	1.03	0.97-1.08	1.03	0.94-1.12	1.02	0.96-1.09
	1 Day		0.99	0.94-1.05	0.98	0.90-1.07	1.00	0.93-1.06
	2 Day		1.02	0.97-1.07	1.03	0.95-1.12	1.00	0.94-1.07
	3 Day		0.99	0.94-1.04	1.03	0.94-1.12	0.97	0.91-1.03
	0 to 2		1.02	0.94-1.10	1.00	0.89-1.13	1.01	0.92-1.12

*Relevant exposure lag according to minimum AIC.

Controlling for holidays and apparent temperature (lag 1).

IQR used is approximately representative of year and season range.

An IQR increase in single pollutants on the day of the attack was associated with a relative risk of 1.12 (RR = 1.12; 95% CI: 1.07, 1.17) for exposure to NO_2_, 1.05 (RR = 1.05; 95% CI: 1.02, 1.08) for exposure to CO, and 1.02 (RR = 1.02; 95% CI: 0.99, 1.05) for PM_2.5_. The variable with the best model fit in the exploratory analysis for ozone was an average of lag 0, 1 and 2 days. The relative risk for the cumulative variable for an IQR increase in ozone was found to be 1.07 (RR = 1.07; 95% CI: 1.03, 1.11). No effect was found for SO_2_.

**Figure 3:**
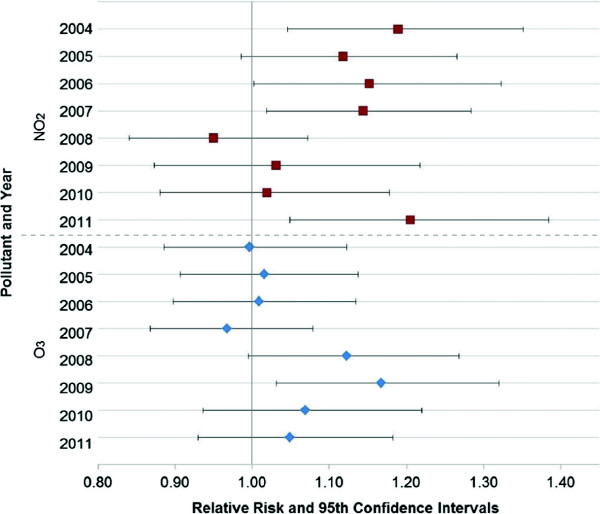
**Change in association between EMS-treated asthma attacks and NO**_**2 **_**and ozone (2004-2011).**

The multi-pollutant pollutant models showing the highest risk were for levels of NO_2_ and ozone. The acute asthma attack risk for these pollutants by year of the study is shown in Figure 3. The adjusted relative risks shown in Figure 3 were obtained from a multi-pollutant model in relation to an increase in the IQR. Controls for apparent temperature and holidays were included in the models. Although the number of cases differ between years, (i.e., there were more cases in 2007 and 2008 than the other years and fewer in 2004 (Table 1)), the pattern indicates that the risk associated with ozone is somewhat inverse that of NO_2_. While at first this may seem logical given that NO_2_ is a component in the formation of ozone, the relationship is not as simply defined. Taken together between the two pollutants, there is no apparent downward trend in the risk of an asthma attack. It is worth noting that ozone and NO_2_ correlations on the daily level were not strong, (r = 0.23), and the monthly patterns differ (see Figure 2).

Regression modeling including the significant associations (i.e., those lags marked with *) discussed above (i.e., all pollutants but SO_2_), and controlling for confounding variables, was used to identify the final multi-pollutant model for the pollutants ozone and NO_2_. Again, based on an IQR level increase in pollution, the relative risk due to ozone is 1.05 (RR = 1.05; 95% CI: 1.00, 1.09) and for NO_2_ is 1.10 (RR = 1.10; 95% CI: 1.05, 1.15). The interaction terms between ozone and NO_2_ or these pollutants with apparent temperature were not significant. The single pollutant risks were dampened slightly compared with the multi-pollutant model. The risk from both pollutants decreased 2% in multi compared with single pollutant models.

### Multi-pollutant model analysis

**Figure 4:**
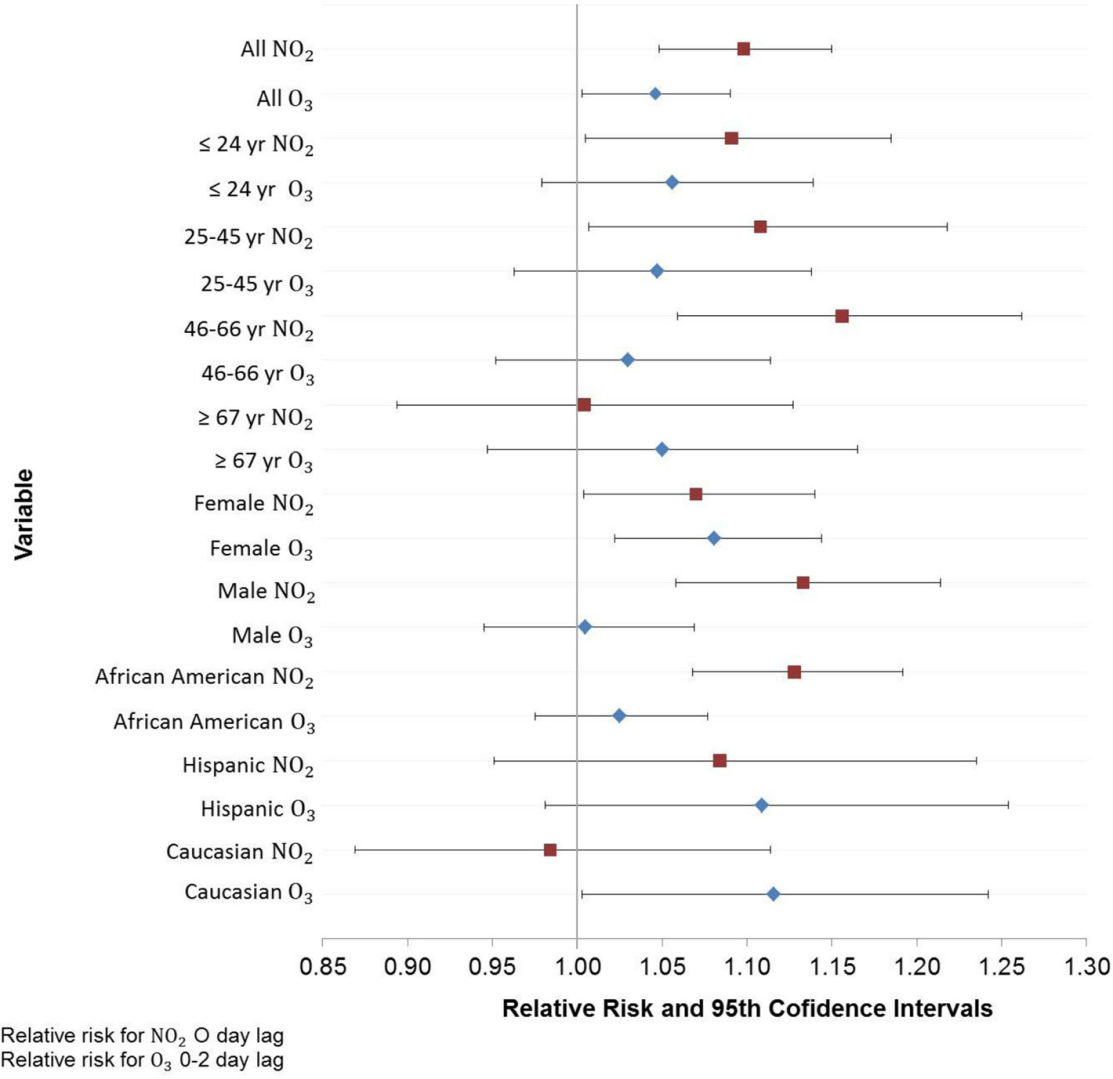
**Change in association between EMS-treated asthma attacks and NO**_**2 **_**and ozone by demographics.**

Results of the multi-pollutant model stratified by age, sex and race groups are shown in Figure 4. Adjusted relative risks shown in Figure 4 were obtained from a multi-pollutant model in relation to an increase in the IQR controlling for apparent temperature and holidays.

The overall multi-pollutant model association was generally stable across age groups (relative risk for an increase in IQR): 24 years and less for ozone and NO_2_, (RR = 1.06; 95% CI: 0.98, 1.14), (RR = 1.09; 95% CI: 1.01, 1.19); 25 to 45 years for ozone and NO_2_, (RR = 1.05; 95% CI: 0.96, 1.14), (RR = 1.11; 95% CI: 1.01, 1.22); 46 to 66 years old for ozone and NO_2_, (RR = 1.03; 95% CI: 0.95, 1.11), (RR = 1.16; 95% CI: 1.06, 1.26) and for 67 years and older for ozone and NO_2_, (RR = 1.05; 95% CI: 0.95, 1.17), (RR = 1.00; 95% CI: 0.89, 1.13). Stratification by sex indicated ozone and NO_2,_ (RR = 1.08; 95% CI: 1.02, 1.14), (RR = 1.07; 95% CI: 1.00, 1.14), relative risk for an increase in IQR, respectively, had a similar effect on females. However, NO_2_ had a stronger effect on males than ozone, (RR = 1.13; 95% CI: 1.06, 1.21) and (RR = 1.01; 95% CI: 0.95, 1.07), relative risk for an increase in IQR respectively. Stratification by race indicated, per increase in IQR, NO_2_ dominated the risk for African Americans (RR = 1.13; 95% CI: 1.07, 1.19) for NO_2_, (RR = 1.03; 95% CI: 0.98, 1.08) for ozone, while ozone dominated the risk for Caucasian (RR = 1.12; 95% CI: 1.00, 1.24) for ozone, (RR = 0.98; 95% CI: 0.87, 1.11) for NO_2_. The risk per IQR for Hispanics was similar between NO_2_ and ozone but slightly shifted toward ozone, (RR = 1.11; 95% CI: 0.98, 1.25) for ozone, (RR = 1.08; 95% CI: 0.95, 1.24) for NO_2_. The lower percentage of EMS calls for all races except African Americans likely impedes useful comparisons between races (see Table 1).

### Analysis by levels of ozone and NO_2_

In a separate analysis, the dataset was divided into bins by ozone and NO_2_ levels over the study period. The segmentation based on level of each pollutant was used to examine the difference in risk with respect to two important factors to consider when constructing guidance for asthma action plans.

**Figure 5:**
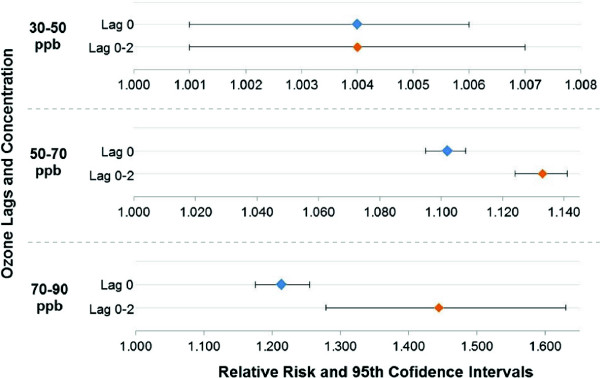
Ozone single pollutant model concentration-risk plot comparing lag 0 day with cumulative lag 0-2 day.

The first important factor is the relevant exposure period. We examine the difference in risk when exposure prevention guidelines are focused on concentrations for the day of the asthma event, to those that include the day of the event and the two previous days. The latter time period is the relevant exposure time for Houston, however current warnings focus only on daily levels of pollutants. Figure 5 shows the concentration-risk plot for the ozone single pollutant model for lag 0 compared with the cumulative effect of lag 0 to 2 days. The results shown in Figure 5 are adjusted for apparent temperature and holidays. Scales differ in the figure for each pollution level. Modeling results indicate that as the ozone concentrations increase, accounting for the cumulative effect of lag 0 to 2 days becomes more important. At the highest bin level of 70 to 90 ppb for the maximum daily 8 hour average concentration, the point estimate risk from the cumulative effect of lag 0 to 2 days is twice as high as the risk from lag 0 day. The risk at this level is also more variable for the cumulative effect of lag 0 to 2 days than the risk from lag 0 day. The finding that cumulative ozone, of up to three days, has a stronger impact than single day past levels has been found by other researchers [46].

**Figure 6:**
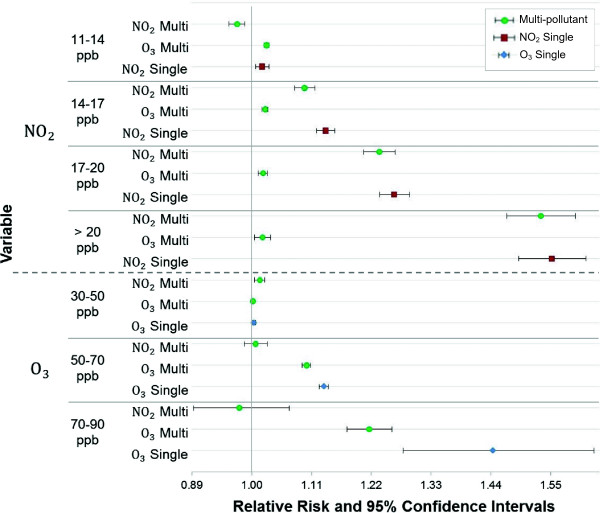
**Concentration-risk plot comparing single pollutant and multi-pollutant models for NO**_**2 **_**and ozone.**

The second issue to consider when constructing asthma action plans regards the city-specific multi-pollutant mixture. We examine the difference in risk when pollutants are considered in isolation compared with a multi-pollutant context. For Houston, the significant multi-pollutant model includes ozone and NO_2_. We found that compared with the single pollutant models of these constituents, the risk attributed to NO_2_ is slightly dampened when ozone is considered, and the risk for ozone is greatly reduced when NO_2_ is considered. Forrest plots of the relative risk are shown in Figure 6 where the upper half of the plot reflects the difference in the risk of single and multi-pollutant models as NO_2_ concentrations increase and the bottom half of the plot reflects these differences when ozone increases.

**Figure 7:**
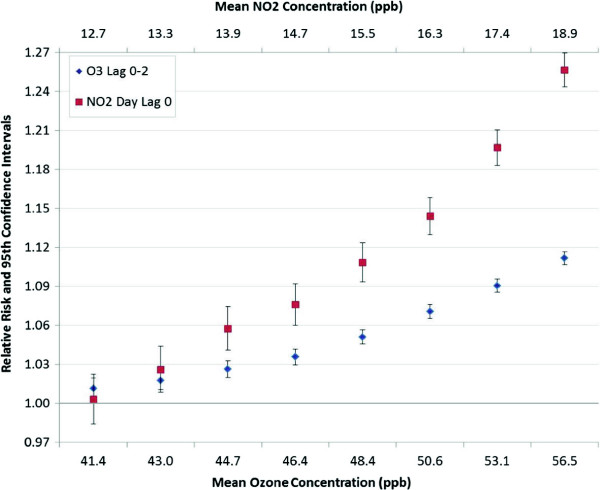
**Forest plots of rolling concentration bins NO**_**2 **_**and ozone multi-pollutant model concentration-risk.**

The results of a multi-pollutant model evaluated for combinations of bins by quartiles of concentrations are shown in Table 4. Quartiles are based on the NO_2_ daily concentrations and the eight-hour highest daily average for ozone. For example the lower right cell of the table contains the risk from exposure to ozone and NO_2_ from the multi-pollutant model run on the segmented data containing only those asthma cases that occurred when both NO_2_ and ozone were in the fourth quartile. This cell also contains the number of days where both ozone and NO_2_ were high (i.e., in the fourth quartile) and the number of asthma cases occurring in that segment. These results indicate that in general the exposure to NO_2_ is associated with a greater risk than ozone, in Houston, and that during the 217 days during the study period when both of the pollutants were simultaneously high (both in quartile 4), there were 952 calls to EMS for asthma attacks requiring albuterol. The relative risk attributed to NO_2_ during that time was 1.44 (RR = 1.44; 95% CI: 1.38, 1.50) and 1.06 (RR = 1.06; 95% CI: 1.04, 1.07) for ozone. The full model indicated that there was no statistically significant interaction between ozone and NO_2_. However when we look at the bin results of Table 4 when NO_2_ is in the fourth quartile, the risk from NO_2_ increases as ozone increases (i.e. the last column in Table 4). Figure 7 is a plot of the risk as the concentrations of ozone and NO_2_ increase.

**Table 4:** Relative risk for multi-pollutants by quartile bins of concentrations during study period

			**Q 1**	**Q 2**	**Q 3**	**Q 4**
			**NO**_2_^*^	**NO**_**2**_	**NO**_**2**_	**NO**_**2**_
			**≤ 7 ppb**	**8-11 ppb**	**11-15 ppb**	**> 15 ppb**
			**Relative Risk and Confidence Interval**			
**Q 1**	**O**_3_^* ^**< 28 ppb**	**Cases**	967	982	609	509
		**Days**	249	232	141	117
		**O**_**3**_	0.93 (0.91-0.94)	0.91 (0.89-0.92)	0.93 (0.92-0.94)	0.93 (0.91-0.95)
		**NO**_**2**_	0.55 (0.51-0.59)	0.84 (0.81-0.86)	0.96 (0.94-0.99)	1.20 (1.17-1.23)
**Q 2**	**O**_**3 **_**28-36 ppb**	**Cases**	737	820	574	705
		**Days**	194	184	122	146
		**O**_**3**_	1.00 (0.99-1.02)	1.00 (0.99-1.01)	1.01 (1.00-1.02)	1.00 (0.98-1.02)
		**NO**_**2**_	0.45 (0.41-0.50)	0.83 (0.80-0.86)	0.96 (0.94-0.99)	1.24 (1.21-1.27)
**Q 3**	**O**_**3 **_**36-48 ppb**	**Cases**	500	972	669	747
		**Days**	131	235	150	152
		**O**_**3**_	1.06 (1.05-1.07)	1.04 (1.03-1.05)	1.02 (1.01-1.03)	1.02 (1.01-1.03)
		**NO**_**2**_	0.56 (0.52-0.61)	0.81 (0.79-0.83)	1.03 (1.00-1.06)	1.27 (1.24-1.30)
**Q 4**	**O**_**3 **_**> 48 ppb**	**Cases**	191	746	1074	952
		**Days**	48	189	245	217
		**O**_**3**_	1.13 (1.10-1.16)	1.09 (1.08-1.11)	1.07 (1.06-1.08)	1.06 (1.04-1.07)
		**NO**_**2**_	0.76 (0.67-0.85)	0.86 (0.83-0.90)	1.12 (1.09-1.15)	1.44 (1.38-1.50)

*O_3_ Day Lag 0 to 2; NO_2_ Day Lag 0.

## Discussion

The results indicate that when the pollutants in Houston were considered together in a multi-pollutant model, two pollutants stood out as triggers for attacks: ozone and NO_2_. Clinical studies indicate that these two pollutants appear to act similarly in triggering an attack because they are both oxidant gases that cause inflammation of the deep lung and respiratory tract [8,9]. Exposure to them may prime eosinophils to subsequent activation by inhaled allergens in atopic patients [47]. The effect of exposure to both pollutants in a mixture has been explored to some degree using bolus-response studies in humans. These studies found that previous continuous exposure to ozone decreases the absorption of a bolus of ozone. This decrease is likely due to depletion of compounds able to absorb ozone. However, the absorption of the ozone bolus increased when there was simultaneous exposure to NO_2_[48]. EPA’s review of studies that examine ozone and NO_2_ binary mixtures concluded that, “very generally, additivity occurred after acute exposure and synergism occurred with prolonged exposure.” While laboratory exposure patterns can’t accurately simulate real-world exposure, findings from the laboratory appear to be consistent with those seen in the population: there is an increase in risk when both pollutants are present, especially at higher concentration. Regardless of the degree of interaction, it is reasonable to expect that exposure to high levels of both pollutants simultaneously increases the risk.

This study found that relative risk in the multi-pollutant model due to an 8 ppb increase in NO_2_ is 1.05 (RR = 1.05; 95% CI: 1.00, 1.09), whereas with a 20 ppb increase in ozone the relative risk is 1.10 (RR = 1.10; 95% CI: 1.05, 1.15). For ozone, the cumulative effect of exposure on the day of the attack and the two days prior pose the greatest risk (0-2 day lag), while for NO_2_ the greatest risk occurs from exposure on the day of the attack. Failing to account for risk, that is attributed to pollutants differently when considered together, especially as concentrations increase, can lead to faulty assumptions regarding which pollutants to attribute the risk.

All age groups below 67 years are at risk from increased levels of the pollution mixture. The risk from NO_2_ exposure appears to increase with increasing age. The risk from NO_2_ is higher for males than females, although more females required EMS treatment for asthma in this study. Ozone and NO_2_ concentrations dip in June and July similar to case numbers. However, in the fall and spring both pollutants can be simultaneously high and case numbers also trend up in this period. The linear dose-response assumption for a plot of the risk as the concentrations of ozone and NO_2_ increase (Figure 7) is a good fit for ozone and a reasonable fit for NO_2_ until very high concentrations. Days with both high ozone and high NO_2_ in Houston can be partially explained by a component of the conceptual model for ozone formation in the Houston-Galveston Area [49]. Land/sea breeze flow reversal occurs when high pressure dominates the area, resulting in light synoptic scale forcing. The light winds and subsidence allow high concentrations of pollutants to accumulate during the night and morning hours, and the land breeze carries the pollutants out over Galveston Bay and into the Gulf of Mexico. During the afternoon, the sea breeze flow reversal carries the ozone back into the city and potentially over freshly emitted NO_2_.

### Comparison with other studies

The association between ambient air pollution and asthma related health effects have been explored by several researchers in single city analyses e.g., [27,28,50-52]. However, reviews and meta-analysis of studies have not found a consistent message [53]. For example, in a review of 19 studies focused on children, exposure to 10 μg/m^3^ of NO_2_, nitrous oxide, and CO were associated with an increased prevalence of asthma ((meta-OR: 1.05, 95% CI: 1.00, 1.11; meta-OR: 1.02, 95% CI: 1.00, 1.04; and meta-OR: 1.06, 95% CI: 1.01, 1.12), SO_2_ was associated with an increased prevalence of wheeze (meta-OR: 1.04, 95% CI: 1.01, 1.07), NO_2_ was associated with an increased incidence of asthma (meta-OR: 1.14, 95% CI: 1.06, 1.24) and particulate matter was associated with an increased incidence of wheeze (meta-OR: 1.05, 95% CI: 1.04, 1.07) but no common thread was found for exposure to ozone [54].

One reason for the inconsistencies may be a result of using different indicators to measure air pollution [55]. Studies in some locations focus on a subset of pollutants because pollutant concentration information is not consistently available. For example, in a study in Detroit ozone was excluded because it was only collected in the warm season and daily PM_2.5_ was imputed from data collected every third day [28]. The number and spatial coverage of monitors measuring pollution is also highly variable. Where the Detroit study used data from four monitors to derive the average pollutant concentration, two were used in a study in Spain [52], 24 for PM_2.5_ and 13 for ozone were used in a study in New York [27], and our study used 9 for PM_2.5_ and 35 for ozone. As discussed previously, differences in results may also be a function of differences between cities (e.g., pollutant mixtures, geography, ethnicity, socioeconomic status, climate, time activity patterns, study cohort including age group and other reasons [28]).

Finally, a direct comparison between the results from the Houston study and other studies is not possible because to our knowledge, this is the first study to examine the association between air pollution and ambulance-treated asthma attacks. The difference in either attack onset or severity a patient experiences requiring the use of an ambulance over traditional emergency department visits is not known.

Still, a comparison of the Houston study results with a meta-analysis and a multi-city study [54,56] was conducted. In the meta-analysis of nineteen studies [54], the relative risk for incidence of asthma associated with NO_2_ exposure, odds ratios converted to the same scale as the Houston study, was 1.11 (RR = 1.11; 95% CI: 1.05, 1.19). This relative risk for NO_2_ is similar to the relative risk of 1.10 found from NO_2_ in the Houston study multi-pollutant model. Recall that for Houston, ozone and NO_2_ are important triggers (RR = 1.05; 95% CI: 1.00, 1.09), (RR = 1.10; 95% CI: 1.05, 1.15) with 20 and 8 ppb increase in ozone and NO_2_, respectively, in a multi-pollutant model. However, the meta-analysis [54], found no risk from ozone exposure whereas the Houston study did.

While in the multi-city study of 14 hospitals in seven cities [56], the relative risk for respiratory related emergency department visits from exposure to ozone was 1.03 (RR converted to Houston scale: 1.03, 95% CI: 1.00, 1.07% per 20 ppb increase). This relative risk from ozone exposure is similar to the ambulance treated relative risk of 1.05 from ozone exposure in Houston. This study found no risk from NO_2_ exposure [56] while the Houston study did.

When the association was found, NO_2_ in the meta-analysis and ozone in the multi-city analysis, the relative risks were of a similar magnitude. Yet, where the Houston study found both ozone and NO_2_ to be of importance, neither the meta-analysis nor the multi-city study found both pollutants to be significant. Studies which examined the association between and asthma and ozone and NO_2_ as co-pollutants found inconsistent results with respect to statistical significance and relevant exposure period e.g., [12,46,50,52,57,58].

## Conclusions

This work was conducted to inform guidance to reduce exposure to air pollution triggered asthma attacks and avert asthma related public health emergencies in a major city with significant air pollution. As discussed previously, the current approach to reduce exposure to air pollution triggered asthma through air pollution alerts based on national single pollutant warning levels may be severely limited and too vague. In the ten years since the National Research Council identified the need to address the effect of mixtures of multi-pollutant that trigger asthma, no new or specific guidance has been established for asthmatics.

The method used here, case cross-over analysis with conditional logistic regression applied to a specific city, was chosen until multi-pollutant statistical modeling methods evolve and are able to dictate specific national guidance for public health intervention. Case cross-over analysis has drawbacks when applied to more than one pollutant at a time (e.g., diminished statistical power as pollutants are added, difficulties identifying higher order interaction, confounding from correlation) [14,16-18]. To preserve power, we focus only on five pollutants, all previously linked to asthma attack triggers. Of those five, the two pollutants, ozone and NO_2_ that stood out most as triggers in the single pollutant model remained in the multi-pollutant model. The contaminant with the third highest relative risk in the single pollutant model, CO, was correlated with NO_2_ (r = 0.74). Fully understanding the confounding between these pollutants is not a concern for this application because an asthma plan tracking NO_2_ would be protective for CO.

These results seen in both the single and multi-pollutant model provide confidence in the conclusion that the asthma related pollutants of concern in Houston can be tracked with ozone and NO_2._ While Houston health care workers have likely been concerned with ozone impacts on asthmatics because Houston ozone levels are above the EPA criteria pollutant standard for ozone, this study provides local quantitative evidence of the link. Since the area NO_2_ levels are below the EPA criteria pollutant standard, the link with this pollutant is new and important information to Houstonians.

Beyond identification of two pollutants of concern that increase the risk of an adverse health effect, important information related to the relevant exposure period prior to triggering an adverse health effect was found (e.g., hours, a day, or extended days). This study concluded that in Houston, the relevant exposure period for NO_2_ is on the order of one day, but the cumulative effect of ozone over a three-day period posed a significantly different and higher risk as concentrations increased compared with the single day risk estimates. This concept of a cumulative effect from ozone is also new and important information for a community member.

On a city-specific level, this analysis provides detailed results that could help prevent attacks by identifying: those individuals in Houston, Texas that may be most at risk of an acute asthma attack requiring EMS treatment triggered from air pollutants; which pollutants trigger the attack, the relevant time period of exposure; and the magnitude of increased risk as concentrations increase.

Asthma action plans in Houston may identify these pollutants as important asthma attack triggers, especially when they are simultaneously warn of the cumulative effect of ozone, and recommend tracking personal sensitivity as pollutants increase, especially for the most at-risk demographics.

## Abbreviations

EPA: Environmental Protection Agency; O_3_: Ozone; NO_2_: Nitrogen dioxide; PM: Particulate matter; SO_2_: Sulfur dioxide; CO: Carbon monoxide; EMS: Emergency medical services; PM2.5: Particulate matter with diameter less than 2.5 microns; TCEQ: Texas Commission on Environmental Quality; AIC: Akaike’s information criterion; IQR: Interquartile range; RR: Relative risk; CI: Confidence intervals; ppb: Parts-per-billion; meta-OR: Meta-analysis odds ratio.

## Competing interests

The authors declare that they have no competing interests.

## Authors’ contributions

LR conducted the exploratory data analysis, the single and multiple pollutant case-crossover analysis, provided interpretation and was the main author of the methodology and results section of the manuscript. KBE conducted the bin analysis, peer reviewed all of the results, provided interpretation, edited the manuscript and authored the remaining sections of the paper. DP conceived the project, developed the health effects database, provided interpretation of the results and edited the manuscript. All authors read and approved the final manuscript.
